# Effect of Gambogic Acid–Loaded Porous-Lipid/PLGA Microbubbles in Combination With Ultrasound-Triggered Microbubble Destruction on Human Glioma

**DOI:** 10.3389/fbioe.2021.711787

**Published:** 2021-09-15

**Authors:** Feng Wang, Lei Dong, Xixi Wei, Yongling Wang, Liansheng Chang, Hongwei Wu, Shuyuan Liu, Yuqiao Chang, Yaling Yin, Xiaoqiu Luo, Xiaojian Jia, Fei Yan, Nana Li

**Affiliations:** ^1^ Henan Key Laboratory of Medical Tissue Regeneration, School of Basic Medical Sciences, Xinxiang Medical University, Xinxiang, China; ^2^ Department of Physiology and Pathophysiology, School of Basic Medical Sciences, Xinxiang Medical University, Xinxiang, China; ^3^ Department of Human Anatomy, Histology and Embryology, School of Basic Medical Sciences, Xinxiang Medical University, Xinxiang, China; ^4^ Department of Chemistry, Xinxiang Medical University, Xinxiang, China; ^5^ Department of Infectious Diseases, The Third Affiliated Hospital of Xinxiang Medical University, Xinxiang Medical University, Xinxiang, China; ^6^ Shenzhen Kangning Hospital and Shenzhen Mental Health Center, Shenzhen, China; ^7^ CAS Key Laboratory of Quantitative Engineering Biology, Shenzhen Institute of Synthetic Biology, Shenzhen Institutes of Advanced Technology, Chinese Academy of Sciences, Shenzhen, China

**Keywords:** ultrasound, gambogic acid, lipid-PLGA microbubbles, ultrasound-targeted microbubble destruction, human glioma

## Abstract

Gambogic acid (GA) is a highly effective antitumor agent, and it is used for the treatment of a wide range of cancers. It is challenging to deliver drugs to the central nervous system due to the inability of GA to cross the blood–brain barrier (BBB). Studies have shown that ultrasound-targeted microbubble destruction can be used for transient and reversible BBB disruption, significantly facilitating intracerebral drug delivery. We first prepared GA–loaded porous-lipid microbubbles (GA porous-lipid/PLGA MBs), and an *in vitro* BBB model was established. The cell viability was detected by CCK-8 assay and flow cytometry. The results indicate that U251 human glioma cells were killed by focused ultrasound (FUS) combined with GA/PLGA microbubbles. FUS combined with GA/PLGA microbubbles was capable of locally and transiently enhancing the permeability of BBB under certain conditions. This conformational change allows the release of GA to extracellular space. This study provides novel targets for the treatment of glioma.

## Introduction

Gliomas mainly occur in middle-aged people, and they are the most malignant primary brain tumors, representing 70% of primary adult malignant brain tumors with a yearly incidence of ∼0.06% (Schulte et al., 2020; Hartanto et al., 2021; Miyake et al., 2021). Currently, the treatment of gliomas mainly includes surgical resection and postsurgical radiotherapy and chemotherapy (Yilmaz et al., 2020; Karschnia et al., 2021; van der Meer et al., 2021). Chemotherapies or chemoradiotherapies can reduce the tumor size and improve the prognosis, but these treatments have significant side effects, especially in the aged patients (Festuccia et al., 2020; Smith-Cohn et al., 2021). Therefore, it is essential to develop safe and effective treatments with fewer side effects. One of these treatment methods is ultrasound (US)-targeted microbubble destruction (UTMD) (Anderson et al., 2021; Zhao et al., 2021). Recently, low-frequency US combined with microbubbles (MBs) has shown potential for tumor therapy. The use of UTMD-mediated drug delivery is a new method that has been applied in many studies, including the treatment of various central nervous system diseases. Selective blood–tumor barrier opening is an effective method to improve the chemotherapeutic efficacy for brain glioma (Anderson et al., 2021).

A large number of studies have indicated that gambogic acid (GA) has good clinical therapeutic effect in a variety of tumors including lung cancer, liver cancer, breast cancer, cervical cancer, nasopharyngeal carcinoma, and gastric cancer (Anderson et al., 2021; Li et al., 2021). Emerging data suggest that GA exhibits the antitumor effect through multiple distinct mechanisms including the inhibition of cell proliferation, influencing telomerase activity and angiogenesis, induction of apoptosis, and cell cycle arrest (Zhang et al., 2020; Chen et al., 2021). The poor water solubility and low bioavailability of GA limit its practical applications. The required concentration (1.5 μmol/L) of GA in the brain is difficult to achieve due to the presence of BBB.

Recently, with the development of ultrasonography, molecular biology, and microbubble contrast agents, the biological effects of low-frequency US combined with MBs have attracted much attention for clinical treatment (Wang and Zheng, 2019; Ingram et al., 2020; Zhang et al., 2021). Low-frequency US combined with MBs has shown potential for tumor therapy (Wang et al., 2021; Zhang et al., 2021). Focused US (FUS) in combination with MBs for BBB disruption (FUS-BBBD) has been established as a promising technique for the delivery of therapeutic agents to a targeted brain location without invasive surgery. The use of MBs, such as US contrast agents, together with US to improve the therapeutic efficacy has a multitude of applications, ranging from enhancing drug penetration through the tissue to opening of the BBB (Ilovitsh et al., 2020; Liao et al., 2020). The continuous improvement of US MBs is the basis of low-frequency US combined with MBs in tumor therapy (Wang and Zheng, 2019). However, conventional lipid-based MBs have poor drug encapsulation efficiency, and polymer-based MBs show a weak capability in contrast imaging and US-triggered drug release. Common phospholipid microbubbles have a good imaging effect *in vivo*, but the drug-loading rate is very low (Reusser et al., 2020). PLGA microbubbles have high drug-loading efficiency, but the imaging effect *in vivo* is poor (Nigam et al., 2019). Yanchen et al. developed a new type of porous-lipid PLGA hybrid MBs (lipid/PLGA MBs) that solved the dilemma of MBs as imaging agents and drug carriers (Chen et al., 2019). Upon receiving US irradiation at an appropriate energy, lipid/PLGA MBs would rapidly oscillate with periodic change in bubble size. The oscillation of lipid/PLGA MBs may produce stable cavitation, which promotes the transmembrane transport of the drug into the cell. FUS combined with lipid/PLGA MBs was capable of locally and transiently enhancing the permeability of BBB under certain parameters (Chen et al., 2019).

When exposed to US, lipid/PLGA MBs oscillate and eventually produce stable cavitation, resulting in temporary pores and increasing cell membrane permeability (Chen and Konofagou, 2014; Black et al., 2016; Liao et al., 2018). Therefore, it is a novel drug-delivery system (DDS) to enhance drug solubility, promote drug accumulation in tumor cells, and achieve “on-demand” drug release, resulting in increased efficacy of brain glioma treatment. Lipid/PLGA MBs have remarkable advantages including higher entrapment efficiency, improved drug profile, economical preparation method, and ease of drug release owing to three-dimensional porous structures (Chen et al., 2019). In the dual emulsion method (W1/O/W2), the initial emulsion phase (W1) is added to a second aqueous phase containing poly(vinyl alcohol) (W2). In this study, we successfully prepared gambogic acid lipid/PLGA MBs (GA lipid/PLGA MBs) carrying GA by this dual emulsion method for the first time. When subjected to US excitation, GA lipid/PLGA MBs expand/contract, generating a localized mechanical impact on cells to induce transient pores on cell membranes. Moreover, FUS with MBs could open the BBB model, as demonstrated in our study. In addition, the GA lipid/PLGA MBs displayed improved metastasis inhibition as well as enhanced tumoricidal benefits compared with free GA.

## Materials and Methods

### Gambogic Acid Lipid/PLGA MBs

PLGA MBs were fabricated using the water/oil/water (W1/O/W2) dual emulsion method. The operating parameters of the ultrasonic cell pulverizer (SCIENTZ-1200E, Ningbo Scientz Biotechnology, Ningbo, China) parameters were as follows: time, 2 min; pulse, 3 seconds on, 3 seconds off; ampl., 40–50%. The aqueous phase (W1) was obtained by dissolving NH_4_HCO_3_ in deionized water. The GA lipid/PLGA hybrids were synthesized using this method. In brief, GA (Shanghai Aladdin Biochemical Technology, Shanghai, China), DSPC (Avanti), and PLGA (Guangzhou Cellcook Biotech, Guangzhou, China) were dissolved in dichloromethane (3 ml) to prepare the oil phase. After the organic solvent evaporates, the lipid chain is prioritized into the hydrophobic interaction of the PLGA shell. Their hydrophilic groups will face the water environment. Along with NH_3_ and CO_2_ gas emissions, reversible reaction to the right occurs and NH_4_HCO_3_ is decomposed. The gases from the particles produce the structure of the entire GA lipid/PLGA MBs. When the dry sample is exposed to the atmosphere and refreshed with air, the PLGA MBs are finally formed. The prepared GA porous-lipid/PLGA MBs were stored at 4°C. The morphology and structure of PLGA MBs were analyzed by transmission electron microscopy (JEM-2100 TEM, JEOL, Tokyo, Japan). The morphology of GA porous-lipid PLGA MBs was analyzed by scanning electron microscopy (SEM, Akishima, Tokyo, Japan). Particle size distribution was measured using a laser diffractometer (LS13320, Beckman-Coulter, United States). GA absorption at 360 nm wavelength was measured using an UV spectrophotometer (GENESYS 10S UV-Vis spectrophotometer; Thermo Scientific, Boston, MA). A multidetection microplate reader (BioTek, United States) was used to detect OD curves of vine acid concentration and calculate the drug-loading and encapsulation efficiency. Drug-loading efficiency was calculated using the following formula: LE (%) = We/Wm×100%. LE represents the drug-loading efficiency of PLGA MBs, We represents the amount encapsulated in the lipid, and Wm represents the total weight of PLGA MBs. Encapsulation efficiency was calculated using the following formula: EN % = (1−Cf/Ct)×100%. EN represents the encapsulation efficiency percentage of drugs in PLGA MBs, Cf is the amount of free drugs, and Ct is the total amount of drugs in PLGA MBs. We tested the stability of GA porous-lipid/PLGA MBs. The particle size and concentration of GA porous-lipid/PLGA MBs were measured at the 0th, 1st, 2nd, 4th, 6th, and 8th day. The concentration of GA porous-lipid/PLGA MBs is expressed by OD_500_.

### Ultrasound Imaging *In Vivo*: PLGA MBs

The PLGA MBs showed a good body US imaging result. B-mode scan ultrasonography was performed using a Sonix SP High-Performance B-Mode system (Ultrasonix, Richmond, BC, Canada). The age of BALB/c mice is 6–7 weeks. All procedures in the animal experiments were conducted in accordance with the guidelines developed by the National Institutes of Health and approved by the Institutional Animal Care and Use Committee of Xinxiang Medical University. Each mouse was injected with 100 μl PLGA MBs suspension via the tail vein. US imaging experiments were carried out at 4 s and 7, 12, 24, and 30 min before and after injection of the suspension. The liver US image in the comparative mode was recorded. Further analysis was performed using a high-frequency US scanning high-resolution B-mode ultrasonography system. High-resolution B-mode ultrasonography of the right liver was performed with a linear frequency of 8 MHz.

### Cell Culture

Human neuroglioma U251 cells (ATCC Stem Cell Library, United States) were grown in the DMEM or MEM (Hyclone, United States) containing 10% fetal bovine serum (FBS). The cells were maintained at 37°C in an incubator provided with 5% CO_2_ and 90% relative humidity. The cells were digested with 0.25% pancreatin (Nanjing KeyGen Biotech, Nanjing, Jiangsu, China) and subcultured at approximately 80% confluence. The cells were counted using a hemocytometer (Fuchs-Rosenthal counting chamber). A cryopreservation solution with 10% DMSO was used for the cryopreservation of these cells. The cell lines were used fewer than 12 times before using fresh stocks.

### Half-Maximal Inhibitory Concentration

First, 100 μl of cell suspension (50,000 cells/ml) was added to each well in a 96-well Corning™ cell culture plate. The plate was placed in an incubator at 37°C with 5% CO_2_ for 24 h culture. The plates were divided into six groups at different concentrations of GA including 0, 0.5, 1.0, 1.5, 2.0, and 3.0 μmol/L, and six complementary holes were set per group. A zero hole of the cell fluid was set only in the cytometry, the blank control hole that does not add drugs. The culture plates were then incubated at 37°C, and the results were observed after 24 h. A total of 10 µl CCK-8 reagent (Beyotime Biotechnology, Shanghai, China) was added to each well for additional 1 h, and the cell viability was measured using the CCK-8 assay. The absorbance at 450 nm was measured using a multidetection microplate reader (BioTek, United States), and the relative cell viability was calculated.

### The CCK-8 Assay for Cell Viability

Cultured cells were inoculated in a 96-well Corning™ cell culture plate at 1.5 × 10^3^ cells/well and divided into four groups, namely, control, GA, GA/PLGA, and FUS + GA/PLGA groups. The supernatant was incubated with GA, GA/PLGA, and FUS + GA/PLGA at a final concentration of 1.5 μmol/L. The plate was placed in an incubator at 37°C with 5% CO_2_ for 24 h culture. FUS was used with the following acoustic parameters: frequency of the transducer, 1.0 MHz; phase, 0.00, cycle: 10.000 k, offset, 0 mV; integral, 1.000 s; ampl., 100 mvpp; time, 1 min. The plates were divided into six groups of different concentrations. A zero hole of the cell fluid was set only in the cytometry, the blank control hole that does not add drugs. The culture plates were then incubated at 37°C, and the results were observed after 24 h. A total of 10 µl CCK-8 reagent was added to each well for additional 1 h, and the cell viability was measured using the CCK-8 assay. The absorbance at 450 nm was measured using a multidetection microplate reader (BioTek, United States), and the relative cell viability was calculated.

Cell viability was determined using the following formula:

Cell viability (%)  =  (A (stimulated)–A (blank)/(A (control)–A (blank)) × 100%.

A (stimulated): absorbance with cells, CCK-8 solutions, and drug solutions.

A (blank): absorbance with the medium and CCK-8 solution without cells.

A (control): absorbance with cells and CCK-8 solution without drug solution.

### Flow Cytometry for the Cell Viability

The cultured cells were inoculated in a 96-well Corning™ cell culture plate at 1.5 × 10^3^ cells/well. Different groups of U251 cells exhibited different treatment factors. The experiments were divided into four groups: control group, GA group, GA/PLGA group, and FUS + GA/PLGA group (the final concentration of GA in the experimental control group was 1.5 μmol/L). The cells were cultured for 24 h at 37°C and 5% CO_2_. FUS was used with the following acoustic parameters: frequency of the transducer, 1.0 MHz; phase, 0.00; cycle, 10.000 k; offset, 0 mV; integral, 1.000 s; ampl.,100 mvpp; time, 1 min (power amplifier, Tanon Technology, Shanghai, China; US signal generator, AFG3000C, Tektronix; US probe, Shanghai Precision & Scientific Instrument, Shanghai, China). The culture plates were then incubated at 37°C, and the results were observed after 24 h. U251 cells (1 × 10^6^ cells) were harvested using trypsin digestion. The sample was evenly split into three tubes. One tube is a blank control tube, and two tubes are single docking tubes. To each single docking tube was added 5 µl Annexin V-FITC or 10 µl PI. The fluorescence intensity was recorded using flow cytometry with FL2 passage (FSC/SSC gate) as the background. Fluorescence compensation was set using single-stained controls, and matching median compensation algorithms were applied. The FACS Calibur flow cytometer was used for cell apoptosis detection, and FlowJo software was used for the analysis of apoptosis (flow cytometer, Miltenyi Biotech).

### Construction of the Blood–Brain Barrier Model

The mouse brain endothelial cell line (bEnd3, ATCC Stem Cell Library, United States) was used to construct a model of BBB *in vitro*. The plate was placed in an incubator at 37°C with 5% CO_2_. Using Transwell cultures, Transwell experiments (MCEP24H48, Millipore, United States) were performed in 24-well Transwell Corning™ cell culture plates (1 × 10^5^ cells per well, 0·4 μm pore size; Costar). Colonies consisting of 30–40 cells were immunofluorescently stained for zonula occludens-1 (ZO-1). The transendothelial electrical resistance (TEER) was measured using an EVOM2 Epithelial Volt–Ohm meter (Millicell Ers-2; EMD Millipore, Billerica, MA, United States). BBB function of the *in vitro* BBB model was quantified from its TEER. The cells reached confluence in 5 days. To evaluate the effect of FUS combined with PLGA MBs on delivery of substances in the *in vitro* BBB model, 0.5 ml NaF solution (10 μg/ml) or liposome solution (1 mg/ml) was added to the donor chamber of the insert. PLGA MBs (5 μl/hole, 1.5 μmol/L) were diluted in the medium and exposed to FUS for 1 min. Taking 100 μl of the lower liquid, the intensity of the fluorescent signal was detected at an excitation wavelength of 360 nm and an emission wavelength of 460 nm.

### Cytotoxicity for Opening the Blood–Brain Barrier Model *In Vitro*

A cell suspension of 100 μl bEnd3 cells (50,000 cells/ml) was added to each well in a 12-well Transwell chambers. On day 3, 100 μl of U251 cell suspension (50,000 cells/ml) was added to each well in a 12-well Corning™ cell culture plate under Transwell chambers. The plate was placed into an incubator at 37°C with 5% CO_2_ for culture of 5 days, and the BBB model was constructed on day 5. On day 5, four processing factors in Transwell chambers were evaluated. The experiment was divided into four groups, control group, GA group, GA/PLGA group, and FUS + GA/PLGA, for US combined with the PLGA MB group (GA final drug concentration in the control and experimental group is 1.5 μmol/L). After the incubator was precultured for 2 h, the Transwell chambers were abandoned. The cells were incubated for 24 h, and the cell viability was assessed using CCK-8 assay.

### Data Analysis

All data were analyzed using SPSS25. The results are expressed as x ± s, where “x” represents the mean values and “s” is the standard deviation. Comparisons between multiple groups were performed by one-way analysis of variance. *p* < 0.05 was treated as statistically significant.

## Results

### Characterization of GA Lipid/PLGA MBs

**FIGURE 1:**
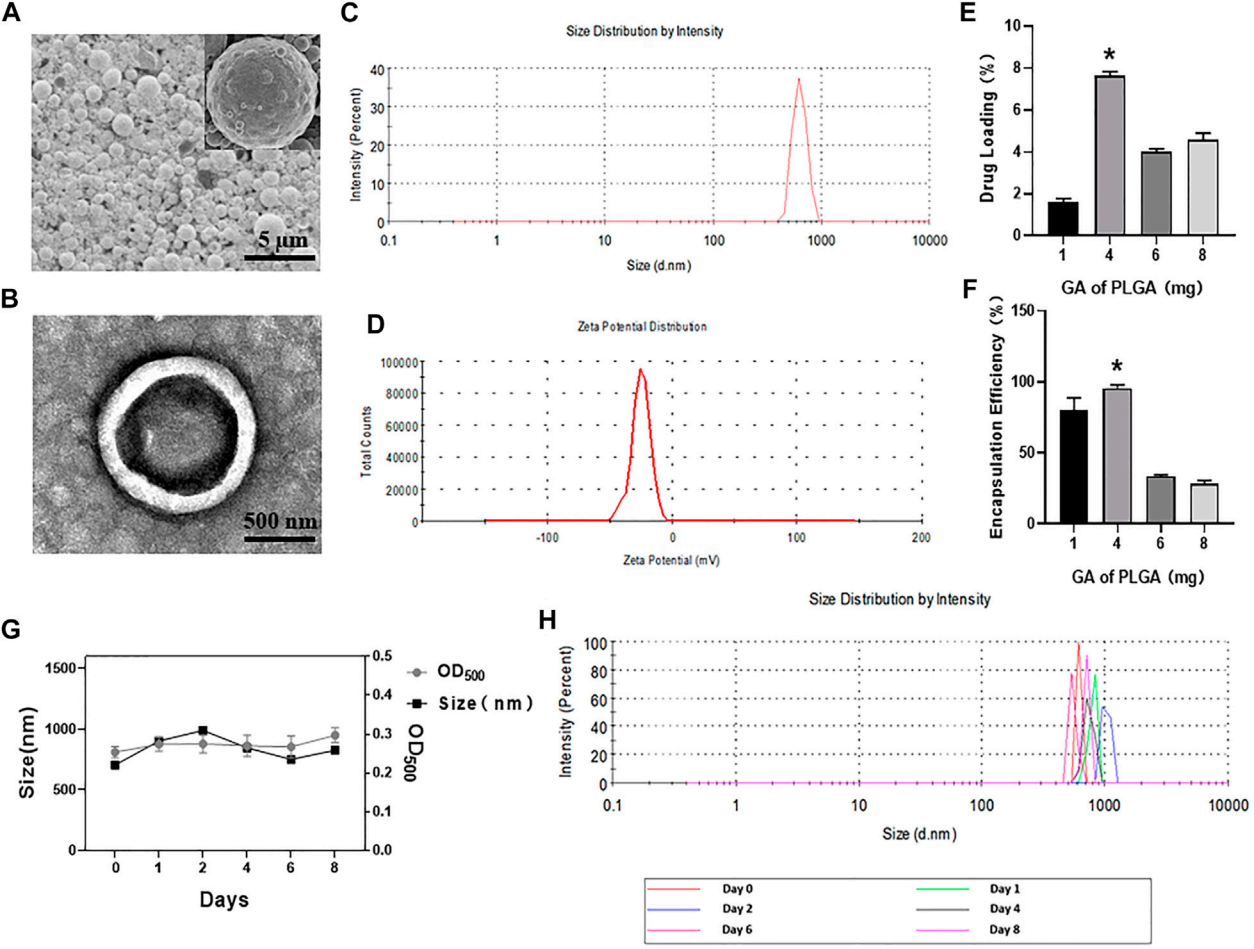
**(A)** SEM images of GA porous-lipid/PLGA MBs. **(B)** TEM images of GA porous-lipid/PLGA MBs. **(C)** Size of GA porous-lipid/PLGA MBs. **(D)** Zeta potential of GA porous-lipid/PLGA MBs. **(E)** Drug loading of GA porous-lipid /PLGA MBs. Compared with other groups, the drug-loading rate corresponding to the 4 mg of GA plus 50 mg of PLGA is 7.65 ± 0.15% (*p < 0.05 versus other three groups). **(F)** Encapsulation efficiency of GA porous-lipid/PLGA MBs. Compared with other groups, the encapsulation efficiency corresponding to the 4 mg of GA plus 50 mg PLGA is 95.57 ± 1.89% (*p < 0.05 versus other three groups). **(G)** The particle size and concentration of GA porous-lipid/PLGA MBs were measured at the 0th, 1st, 2nd, 4th, 6th, and 8th day. The concentration of GA porous-lipid/PLGA MBs is expressed by OD_500_. **(H)** The particle size distribution of GA porous-lipid/PLGA MBs on the 0th, 1st, 2nd, 4th, 6th, and 8th day.

PLGA MBs are spherical, uniformly dispersed and have good stability (Figures 1A,B,G,H). The mean particle size was 851.36 ± 77.01 nm (Figure 1C). The mean potential was –4.55 ± 1.39 mV (Figure 1D). The OD of the GA standard was plotted on the abscissa, and the concentration corresponding to each OD was plotted on the ordinate axis (y = 0.0034x − 0.0007, *R* ^2^ = 0.9985). Then, concentrations of 1, 4, 6, and 8 mg of GA per 50 mg PLGA and 2.5 mg DSPC raw materials were used. PLGA MBs were divided into four groups, and the encapsulation rate and drug-loading efficiency were calculated. Compared with the other three groups, the encapsulation rate of 4 mg of GA added to 2.5 mg of DSPC and 50 mg of PLGA was 95.57 ± 1.89% and the drug-loading rate of it was 7.65 ± 0.15% (*p < 0.05, Figures 1E,F). Figure 1 shows the actual drug-loading rate, encapsulation efficiency, mean particle diameter, and particle size distribution of PLGA MBs.

### *In Vivo* Ultrasound Imaging of PLGA MBs

**FIGURE 2:**
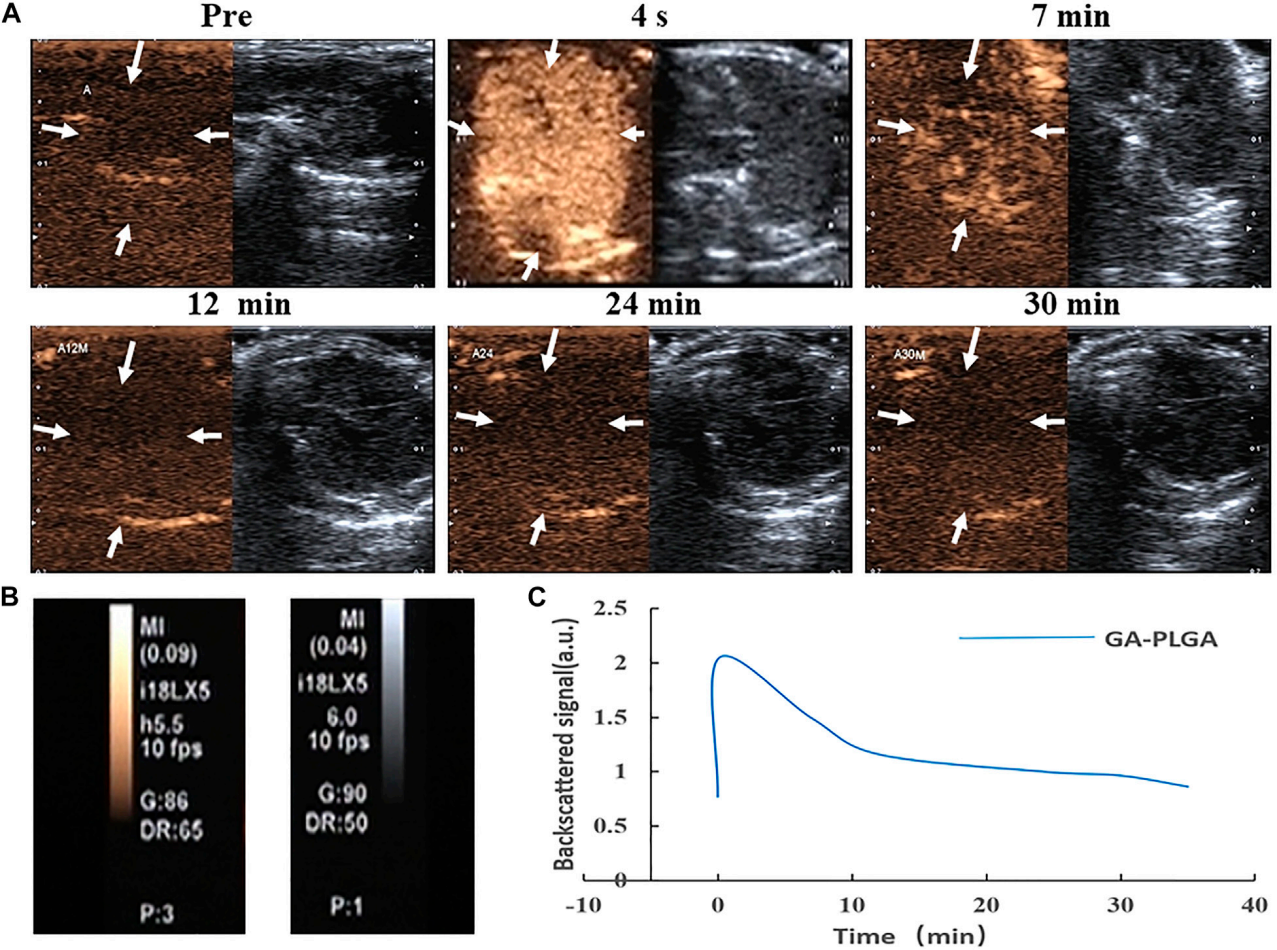
**(A, B)** Ultrasonic images of GA porous-lipid/PLGA MBs. **(C)** Changes in signal strength in the liver. After injection of GA porous-lipid/PLGA MBs, the liver imaging was rapidly enhanced, the signal gradually decreasing after 7 min and completely disappearing after 30 min.

After the tail vein injection of PLGA MBs, the mice were subjected to US imaging experiments at different time intervals (Figure 2A). The liver US image in the comparative mode was recorded. Further analysis was performed using a high-frequency US scanning high-resolution B-mode ultrasonography system. High-resolution B-mode ultrasonography of the right liver was performed with a linear frequency of 8 MHz (Figure 2B). Before the PLGA MB injection, the liver shows a weak ultrasonic contrast signal. After the tail vein injection of PLGA MBs, the liver imaging immediately brightened. This indicates that the PLGA MBs exhibit high efficiency in the ultrasonic examination of the liver. The corresponding quantitative analysis showed that ultrasonic signal strength reached the peak in 4 s, and the enhancement effect lasted for nearly 7 min. After 12 min of injection, the ultrasonic signal strength decreased and completely disappeared after 30 min (Figure 2C). Thus, PLGA MBs exhibit a good body US imaging performance (Figure 2).

### Half-Maximal Inhibitory Concentration of GA for the U251 Cell Line

**FIGURE 3:**
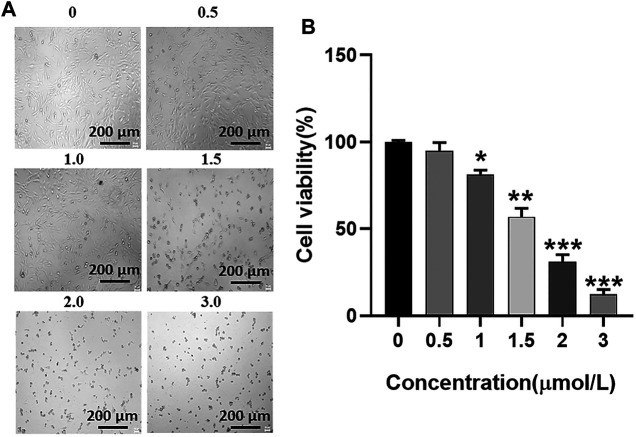
**(A)** Growth status of U251 cells. As the concentration of GA increases, the survival state of the U251 cell line gradually deteriorates. **(B)** Cell viability of U251 cells. After different concentrations of GA were incubated with U251 cells, cell viability of each group was detected by the CCK-8 reagent. When the GA concentration is 1.5 μmol/L, the cell viability is 56.99 ± 3.95% (**p < 0.01 versus “0 μmol/L” group).

U251 cells were incubated with GA at a range from 0 μmol/L to 3 μmol/L for 24 h, and the survival status of U251 cells was observed under the microscope (Figure 3A). The CCK-8 assay was used to detect the cell viability of U251 cells at 24 h after incubation with GA. The cell viability of U251 cells with different concentrations of GA was as follows: 95.00 ± 3.95%, 56.99 ± 3.95%, 31.25 ± 3.24%, and 12.25 ± 2.29% (Figure 3B). The half-maximal inhibitory concentration of GA for the U251 cell line was 1.5 μmol/L (Figure 3).

### Evaluation of Inhibitory Effect of Focused Ultrasound Combined With PLGA MBs on U251 Cells by the CCK-8 Assay

**FIGURE 4:**
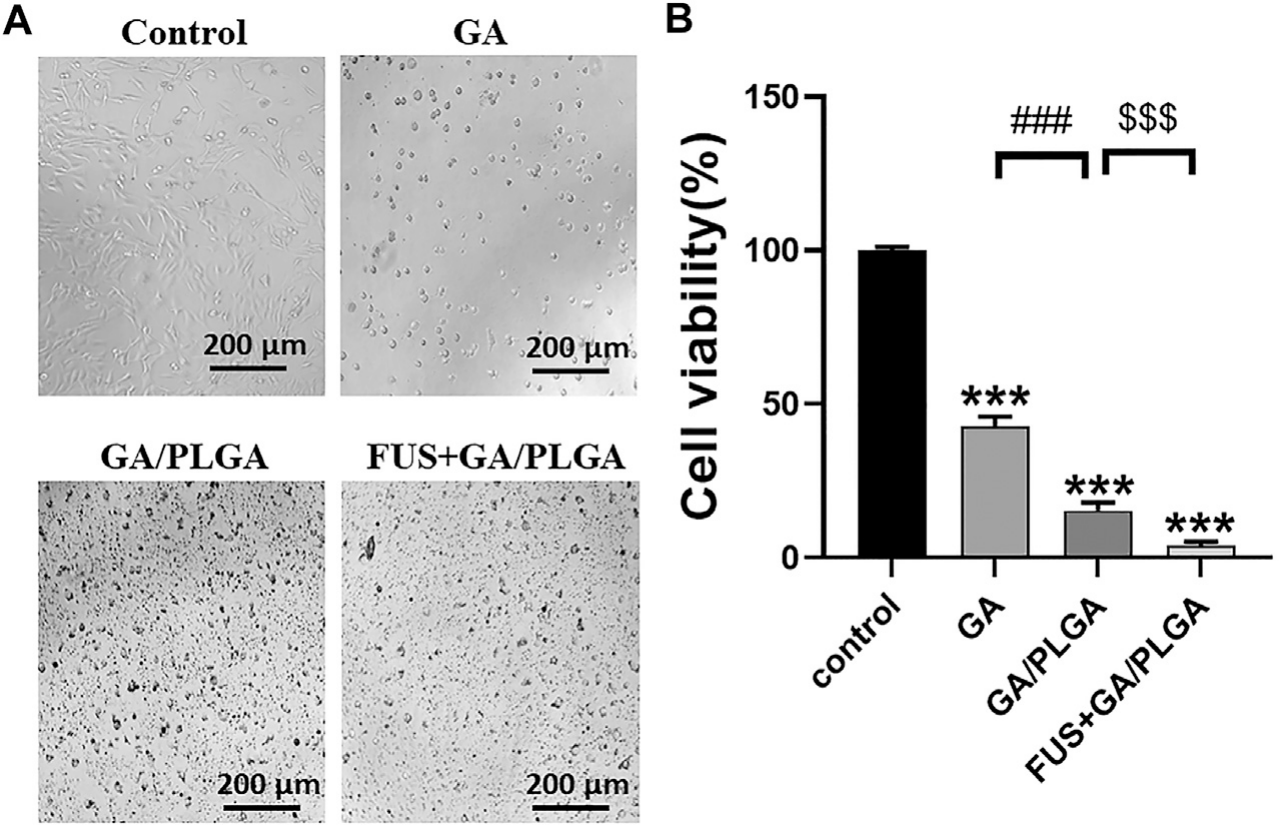
**(A)** After four different treatments for 24 h, the survival state of U251 cells in each group was observed under a microscope. **(B)** The cell viability of U251 cells in each group was detected by the CCK-8 reagent. The cell viability of the FUS + GA/PLGA group was 3.85 ± 0.67% (***p < 0.001 versus the control group and ^$$$^ *p* < 0.001 versus the GA/PLGA group).

U251 cells were treated with four different treatments for 24 h, and the survival status of U251 cells in each group was observed under the microscope (Figure 4A). The CCK-8 assay was used to detect the cell viability of U251 cells at 24 h after incubation. Compared with the control group, the cell viability of U251 cells in the three experimental groups was as follows: 42.87 ± 2.74%, 15.28 ± 2.33%, and 3.85 ± 0.67% (Figure 4B). A comparison of GA, GA/PLGA, and FUS + GA/PLGA groups with the control groups showed a significant statistical difference (***p < 0.001). The results show that these three processing methods have good inhibitory effect on U251 cells. Compared to the GA/PLGA group, the GA group showed a significant statistical difference (^###^ *p* < 0.001). The results show that GA/PLGA was easily phagocytized by U251 cells and it inhibited the growth of U251 cells relative to GA. Compared to the GA/PLGA group, the FUS + GA/PLGA group showed a significant statistical difference (^$$$^ *p* < 0.001). Figure 4 shows that FUS combined with GA/PLGA made GA enter the U251 cells easily, thus promoting a stronger inhibition of U251 cell growth.

### Evaluation of Inhibitory Effect of FUS + GA/PLGA on U251 Cells by Flow Cytometry

**FIGURE 5:**
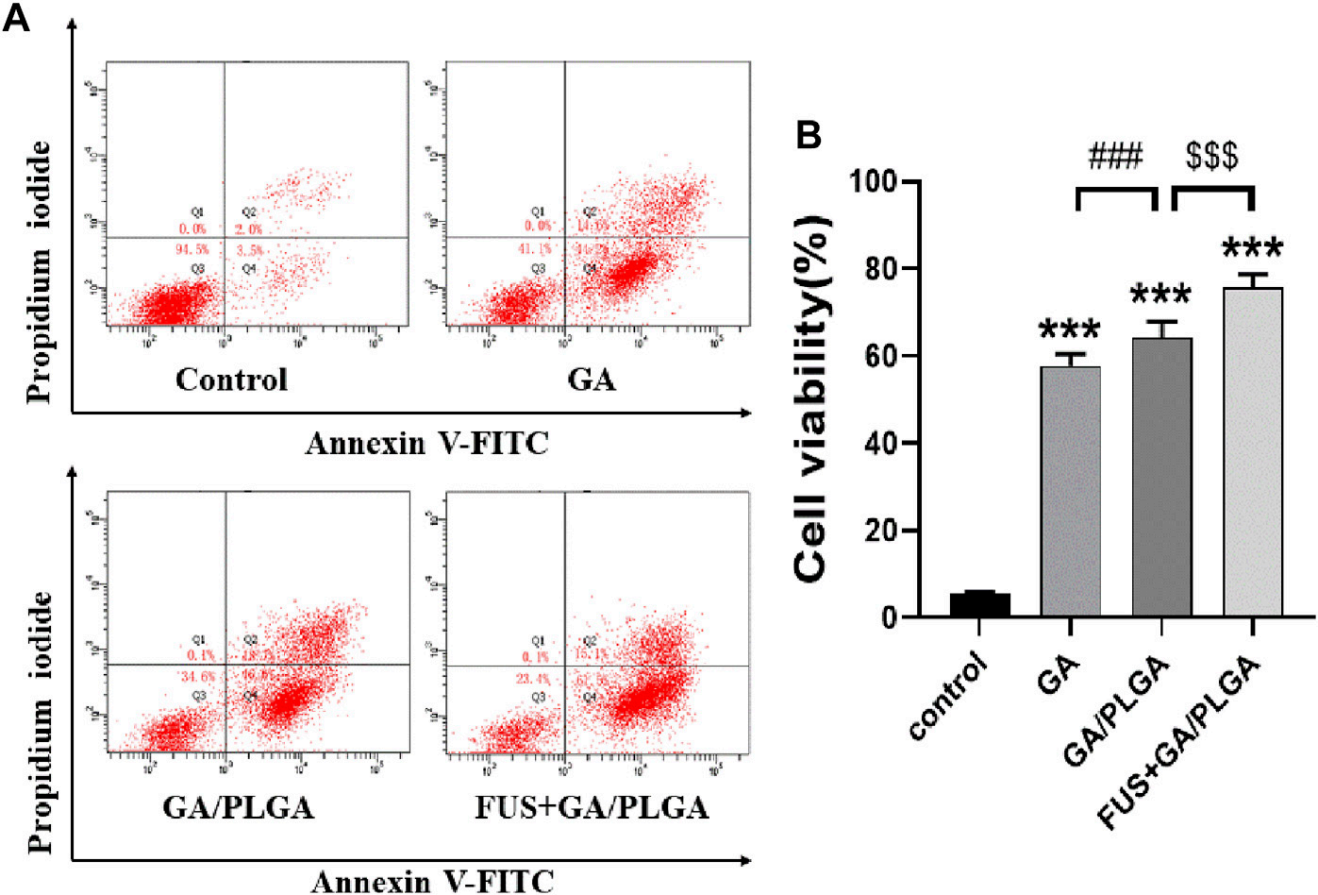
**(A)** The apoptosis test of U251 cells. **(B)** The apoptosis rate of U251 cells. U251 cells were subjected to different treatments for 24 h; flow cytometry detected apoptotic rates in each group. The apoptotic rate of U251 cells in the FUS + GA/PLGA group was 75.97 ± 0.49% (***p < 0.001 versus the control group and ^$$$^ *p* < 0.001 versus the GA/PLGA group).

The apoptosis rates of each group of cells obtained by flow apoptosis are as follows: 5.57 ± 0.17%, 58.77 ± 0.12%, 65.57 ± 0.46%, and 75.97 ± 0.49% (Figures 5A,B). A comparison of the GA, GA/PLGA, and FUS + GA/PLGA groups with the control groups showed a significant statistical difference (***p < 0.001). The results show that these three processing methods can cause apoptosis in U251 cells. Compared to the GA/PLGA group, the GA group showed a significant statistical difference (^#^ *p* < 0.05). The results show that compared with GA, GA/PLGA is more likely to cause apoptosis in U251 cells. Compared to the GA/PLGA group, the FUS + GA/PLGA group showed a significant statistical difference (^$$^ *p* < 0.01). The results show that compared with GA/PLGA, FUS combined with GA/PLGA is more likely to cause apoptosis in U251 cells (Figure 5).

### The BBB Model Opened by *In Vitro* Focused Ultrasound Combined With MBs or GA/PLGA MBs

**FIGURE 6:**
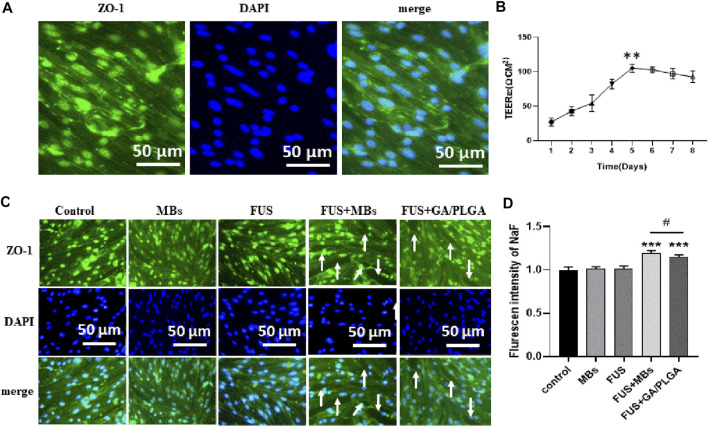
**(A)** The *in vitro* BBB model. Blue indicates the nucleus and green indicates the ZO-1 tight junction protein. **(B)** Resistance change of the *in vitro* BBB model. The resistance value of the BBB model was 105.12 ± 4.92 Ω.CM^2^ on the fifth day (**p < 0.01 versus the other group). **(C)** L (Alexa Fluor488) fluorescent secondary antibody labeled the ZO-1 tight junction protein. DAPI labeled the nucleus. FUS combined with lipid MBs or GA/PLGA MBs could open the BBB model, and the white arrow indicates that the tight junction part was already open. **(D)** Florescent intensity of NaF in the FUS + MBs group was 1.19 ± 0.02 (***p < 0.001 versus the control group). Florescent intensity of NaF in the FUS + GA/PLGA group was 1.19 ± 0.02 (***p < 0.001 versus the control group and ^#^ *p* < 0.05 versus the FUS + MBs group).

The bEnd3 cells in Transwell chambers correspond to 12-well plates (50,000 /ml bEnd3 cells in Transwell chambers). The resistance of the cross-well film in the small chamber was measured continuously from the 1st day to the 8th day (Ω.CM_2_) as follows: 27.12 ± 5.05, 42.95 ± 5.53, 54.26 ± 10.36, 82.52 ± 5.87, 105.12 ± 4.92, 102.86 ± 3.74, 97.21 ± 6.39, and 92.69 ± 7.14. On day 5, the resistance is the highest and it has statistical differences with the resistance corresponding to other days (**p < 0.01). The results show that the BBB model was successfully constructed (Figure 6B). Tight junction protein ZO-1 is stained with anti-ZO-1 antibody, and DAPI staining of the endothelial cell nucleus can be observed in the *in vitro* BBB model (Figure 6A). We proved that FUS combined with MBs or GA/PLGA MBs could open BBB models (Figure 6C). The fluorescent signal values corresponding to the lower-chamber liquid in the control group, GA group, GA/PLGA group, FUS + MBS group, and the FUS + GA/PLGA MBs group are as follows: 1.00 ± 0.03, 1.01 ± 0.02, 1.01 ± 0.03, 1.19 ± 0.02, and 1.14 ± 0.02. Compared with the other groups, the fluorescence signal value of FUS + MBs or GA/PLGA MBs group showed statistical differences (***p < 0.001) (Figure 6D). The results show that FUS combined with MBs or GA/PLGA MBs could open BBB models (Figure 6).

### Inhibition Effect on Glioma Using Focused Ultrasound Combined With PLGA MBs

**FIGURE 7:**
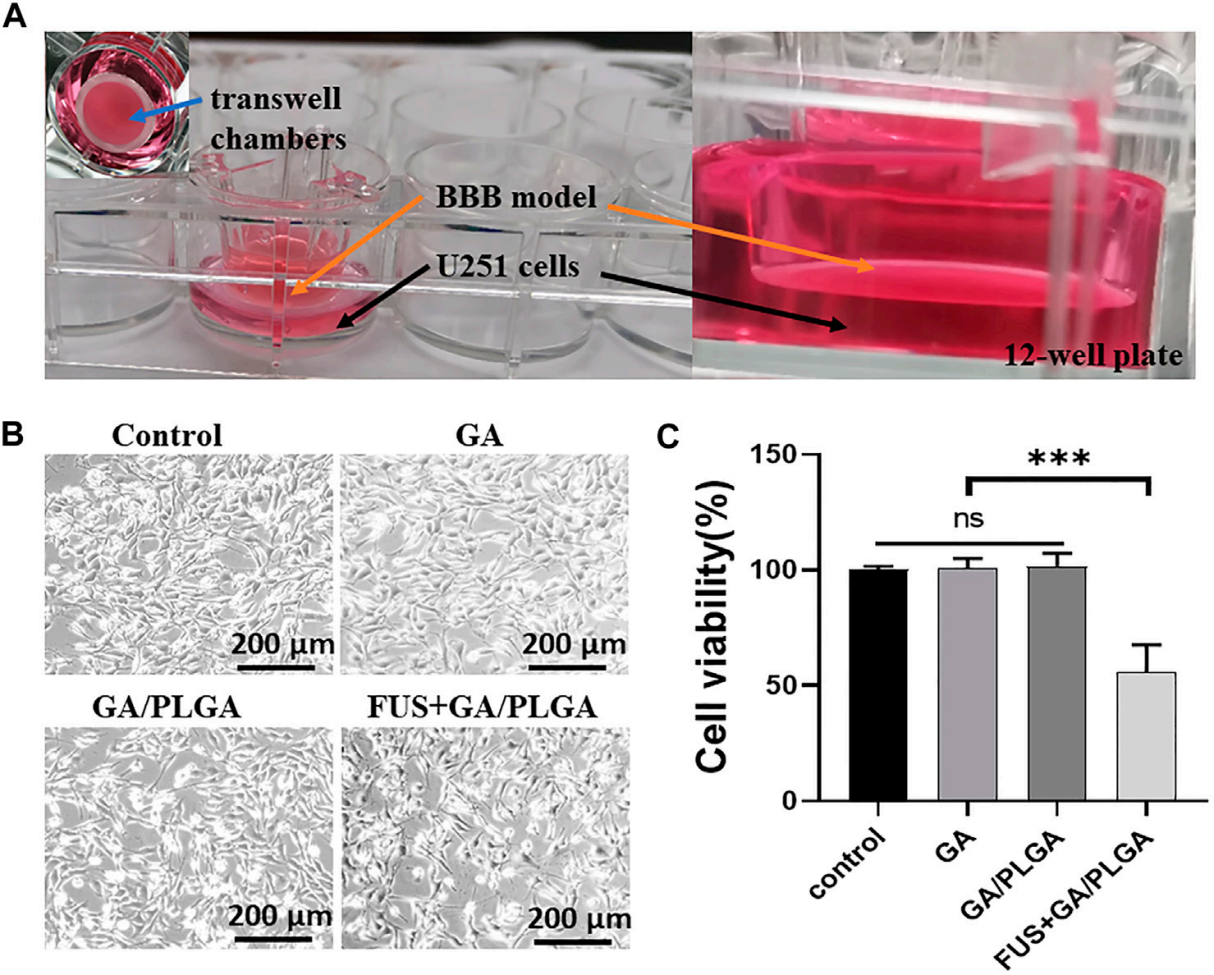
**(A)** Construction of the *in vitro* BBB model. bEnd3 cells were used to build the BBB model. **(B)** After four different treatments for 24 h, the survival state of U251 cells in each group was observed under a microscope. **(C)** The cell viability of U251 cells in each group was detected by the CCK-8 reagent. The cell viability of the FUS + GA/PLGA group was 55.83 ± 10.99% (***p < 0.001 versus the other group).

The bEnd3 cells in Transwell chambers correspond to 12-well plates (50,000/ml bEnd3 cells in Transwell chambers) (Figure 7A). Five days later, the experiments were divided into four groups as follows: control group, GA group, GA/PLGA group, and FUS + GA/PLGA group. After 24 h of different treatments, the survival status of U251 cells in each group was observed under a microscope (Figure 7B). Compared with the control group, the cell viability of U251 cells in the three experimental groups was as follows: 98.61 ± 2.29%, 99.01 ± 4.67%, and 55.83 ± 10.99%. Compared with the cell viability of other groups, the FUS + GA/PLGA group showed statistical differences (*p < 0.05) (Figure 7C).

## Discussion

Mahmount Youns et al. evaluated the anticancer activity of GA against pancreatic cancer cells in a set of different differentiation stages (Youns et al., 2018). In addition, a total-genome transcription map study was carried out to determine the possible factors to adjust the antitumor effect of GA on pancreatic cancer cells. The results of expression analysis showed that vine acid treatment specifically affects the pancreatic cancer signal transduction pathways (Youns et al., 2018). Kaizhao et al. showed that GA had significant inhibitory effects on the primary lung metastasis of A549 cells. GA was developed as a candidate drug with treatment potential for treating tumor invasion and metastasis (Zhao et al., 2017). MBs have recently become a desired US angiography method and drug-delivery carrier. However, conventional lipid-based MBs have poor pharmaceutical encapsulation efficiency, and the polymer-based MBs exhibit weaker capabilities in the drug release of imaging and ultrasonic triggers.

Yao et al. shows that the BBB opens with nonlinear bubble oscillation when the bubble diameter is similar to the capillary diameter and with inertial cavitation when it is not. The bubble may thus have to be in contact with the capillary wall to induce BBB opening without IC. BBB opening was shown capable of being induced safely with nonlinear bubble oscillation at the pressure threshold, and its volume was highly dependent on both the acoustic pressure and bubble diameter (Tung et al., 2011). Yanchen et al. developed a new type of PLGA MBs (lipid/PLGA MBs), which solves the problem of MBs when used as an imager and drug carrier (Chen et al., 2019). BBB reduced the efficiency of nanoparticles entering the brain tissue. Broadening the tight junction of the BBB by FUS provides a promising method to enhance the entry of nanoparticles into the brain tissue. However, the effect of opening the tight junction of the BBB through FUS alone is not obvious. Compared with nanoparticles, the lipid/PLGA MBs shell has better elasticity. The effect of steady-state cavitation is better when lipid/PLGA MBs are combined with FUS. The openness of BBB is more obvious. At the same time, under the action of FUS, lipid/PLGA MBs produce particles less than 100 nm, which can better pass through the open BBB (Conti et al., 2019; Ohta et al., 2020).

We used new PLGA MBs as the carrier. PLGA MBs carrying GA were prepared through water/oil/water (W1/O/W2) dual emulsion. Then, the GA/PLGA MBs were characterized. *In vivo* experiments were conducted, and the liver US image was recorded in a comparative mode. Further analysis was performed using a high-frequency US scanning system, and the US imaging effect on the body was detected. In the *in vitro* experiment, when the vinegar concentration was 1.5 μmol/L, the U251 cell viability was around 50%. Cell viability or apoptosis was examined using the CCK-8 reagent, and flow cytometry was conducted for four different treatment groups such as the control group, GA group, GA/PLGA group, and FUS + GA/PLGA group. The results show that the FUS + GA/PLGA group has the strongest inhibitory effect on U251 cells. bEnd3 cells were used to successfully build *in vitro* BBB models. We proved that FUS combined with PLGA MBs could open the BBB model. GA was better distributed in the U251 region and inhibited its growth.

In addition, passive cavitation detection (PCD) is a method proposed to monitor the microbubble activity during US exposure (Sun et al., 2015; Novell et al., 2020), which is frequently now used in clinical trials (Pouliopoulos et al., 2020). In the next step, we plan to detect the GA/PLGA microbubbles activity *in vivo* by using PCD.

## Conclusion

PLGA MBs carrying GA were prepared through water/oil/water (W1/O/W2) dual emulsion. PLGA MBs showed a good liver US imaging effect. In addition, FUS combined with PLGA MBs exhibited significant inhibitory effects on the U251 cell line. We proved that the FUS combined with PLGA MBs could open the BBB model. GA was better distributed in the U251 region and inhibited its growth. Therefore, the combined method is expected to be applied in the treatment of human glioma, further improving the therapeutic effect of GA on human glioma.

## Data Availability

The raw data supporting the conclusion of this article will be made available by the authors, without undue reservation.
